# Genomic instability in human cancer: Molecular insights and opportunities for therapeutic attack and prevention through diet and nutrition

**DOI:** 10.1016/j.semcancer.2015.03.005

**Published:** 2015-12

**Authors:** Lynnette R. Ferguson, Helen Chen, Andrew R. Collins, Marisa Connell, Giovanna Damia, Santanu Dasgupta, Meenakshi Malhotra, Alan K. Meeker, Amedeo Amedei, Amr Amin, S. Salman Ashraf, Katia Aquilano, Asfar S. Azmi, Dipita Bhakta, Alan Bilsland, Chandra S. Boosani, Sophie Chen, Maria Rosa Ciriolo, Hiromasa Fujii, Gunjan Guha, Dorota Halicka, William G. Helferich, W. Nicol Keith, Sulma I. Mohammed, Elena Niccolai, Xujuan Yang, Kanya Honoki, Virginia R. Parslow, Satya Prakash, Sarallah Rezazadeh, Rodney E. Shackelford, David Sidransky, Phuoc T. Tran, Eddy S. Yang, Christopher A. Maxwell

**Affiliations:** a Discipline of Nutrition, University of Auckland, Auckland, New Zealand; b Department of Pediatrics, University of British Columbia, Michael Cuccione Childhood Cancer Research Program, Child and Family Research Institute, Vancouver, Canada; c Department of Nutrition, Faculty of Medicine, University of Oslo, Oslo, Norway; d Department of Oncology, Instituti di Ricovero e Cura a Carattere Scientifico-Istituto di Ricerche Farmacologiche Mario Negri, Milan, Italy; e Department of Cellular and Molecular Biology, The University of Texas Health Science Center at Tyler, Tyler, United States; f School of Pharmacy, University College Cork, Cork, Ireland; g Department of Pathology, Johns Hopkins University School of Medicine, Baltimore, United States; h Department of Experimental and Clinical Medicine, University of Florence, Florence, Italy; i Department of Biology, College of Science, United Arab Emirates University, Al Ain, United Arab Emirates; j Faculty of Science, Cairo University, Cairo, Egypt; k Department of Chemistry, College of Science, United Arab Emirates University, Al Ain, United Arab Emirates; l Department of Biology, Università di Roma Tor Vergata, Rome, Italy; m Department of Biology, University of Rochester, Rochester, United States; n School of Chemical and BioTechnology, SASTRA University, Thanjavur, Tamil Nadu, India; o Institute of Cancer Sciences, University of Glasgow, Glasgow, United Kingdom; p Department of BioMedical Sciences, Creighton University, Omaha, NE, United States; q Department of Research & Development, Ovarian and Prostate Cancer Research Trust Laboratory, Guildford, Surrey, United Kingdom; r Department of Orthopaedic Surgery, Nara Medical University, Kashihara, Nara, Japan; s New York Medical College, Valhalla, NY, United States; t Department of Food Science and Human Nutrition, University of Illinois at Urbana-Champaign, Champaign, IL, United States; u Department of Comparative Pathobiology and Purdue University Center for Cancer Research, Purdue University, West Lafayette, IN, United States; v Department of Pathology, Louisiana State University Health Shreveport, Shreveport, LA, United States; w Department of Otolaryngology-Head and Neck Surgery, Johns Hopkins University School of Medicine, Baltimore, MD, United States; x Departments of Radiation Oncology & Molecular Radiation Sciences, Oncology and Urology, The Sidney Kimmel Comprehensive Cancer Center, Johns Hopkins School of Medicine, Baltimore, MD, United States; y Department of Radiation Oncology, University of Alabama at Birmingham School of Medicine, Birmingham, AL, United States

**Keywords:** Genomic instability, Cancer therapy, Cancer prevention, DNA damage, Nutraceutical

## Abstract

Genomic instability can initiate cancer, augment progression, and influence the overall prognosis of the affected patient. Genomic instability arises from many different pathways, such as telomere damage, centrosome amplification, epigenetic modifications, and DNA damage from endogenous and exogenous sources, and can be perpetuating, or limiting, through the induction of mutations or aneuploidy, both enabling and catastrophic. Many cancer treatments induce DNA damage to impair cell division on a global scale but it is accepted that personalized treatments, those that are tailored to the particular patient and type of cancer, must also be developed. In this review, we detail the mechanisms from which genomic instability arises and can lead to cancer, as well as treatments and measures that prevent genomic instability or take advantage of the cellular defects caused by genomic instability. In particular, we identify and discuss five priority targets against genomic instability: (1) prevention of DNA damage; (2) enhancement of DNA repair; (3) targeting deficient DNA repair; (4) impairing centrosome clustering; and, (5) inhibition of telomerase activity. Moreover, we highlight vitamin D and B, selenium, carotenoids, PARP inhibitors, resveratrol, and isothiocyanates as priority approaches against genomic instability. The prioritized target sites and approaches were cross validated to identify potential synergistic effects on a number of important areas of cancer biology.

## 1 Cellular mechanisms that prevent or promote genomic instability

Genomic instability plays critical roles in both cancer initiation and progression. This instability can manifest itself genetically on several different levels, ranging from simple deoxyribonucleic acid (DNA) sequence changes to structural and numerical abnormalities at the chromosomal level. This section will briefly outline the mechanisms that maintain the stability of nuclear and mitochondrial DNA and how these mechanisms may become corrupted in cancer cells.

### 1.1 Telomeres foster chromosomal stability and can inhibit or promote malignant transformation

The chromosome stabilizing role of intact telomeres was recognized as early as the 1930s from independent research by McClintock [1] and Muller [2] and more recent work has further strengthened the connection between telomere dysfunction and chromosomal instability (CIN) [3], [4]. Telomeres, which are located at the ends of each chromosome, consist of approximately 5–10 kbp of specialized, tandem repeat, noncoding DNA complexed with a variety of telomere associated proteins [5], [6]. These elements create a protective cap that prevents the recognition of the chromosomal termini as DNA double strand breaks (DSBs) and their consequent aberrant repair via nonhomologous end joining (NHEJ) or homologous recombination (HR) [7], [8], [9], [10]. Due to the inability of DNA polymerase to fully replicate the ends of linear DNA molecules, in the absence of compensatory mechanisms, telomeric DNA is lost at the rate of approximately 100 base pairs (bp) per telomere per cell division [11], [12], [13], [14], [15], [16], [17]. In normal somatic cells, this telomere erosion is used by the cell to monitor its division history, with moderate telomere shortening triggering either irreversible cell cycle arrest, termed replicative senescence, or apoptosis [18], [19], [20], [21]. This block to continued proliferation is thought to have evolved to prevent the development of cancer in long-lived organisms by restricting the uncontrolled outgrowth of transformed cell clones, and also by preventing further telomere erosion which would accompany such abnormal growth and eventually destabilize the telomeres leading to CIN [13], [22].

A current popular model for the involvement of telomere shortening in carcinogenesis posits that increasing numbers of cells experience telomere shortening as a person ages, which increases the pool size of cells that are in danger of experiencing eventual telomere dysfunction and prooncogenic CIN. In the vast majority of such cells, the senescence and apoptotic blocks are strictly enforced [23], [24], [25], [26], [27], [28]. However, this process eventually fails in rare cells which continue to replicate and eventually experience CIN due to critical telomere shortening [15], [29], [30], [31], [32], [33], [34], [35], [36], [37]. Notably, such cells may be more tolerant of rampant genomic instability due to their previous abrogation of the tumor suppressive telomere length checkpoints. However, if left unchecked, this instability will eventually reach lethal levels in the transforming cells, thereby presenting a second block to the development of cancer [37], [38], [39], [40]. This escalating telomere driven CIN creates a strong selective pressure for telomere maintenance in incipient cancer cell populations; a problem that is solved in one of two ways: activation of telomerase or alternative lengthening of telomeres (ALT). In the majority of human cancers, the telomere specific reverse transcriptase telomerase, which is stringently repressed in normal somatic cells, is activated, thereby restabilizing the telomeres, although cancer telomeres on average seem to remain very short [32], [41], [42], [43], [44], [45]. Whereas most cancers use telomerase to maintain telomere length, a significant minority of cancers (typically non-carcinomas) utilize ALT, a telomerase independent, homologous recombination based mechanism [46], [47], [48]. This mode of telomere maintenance results in extreme telomere length heterogeneity and, interestingly, better patient survival compared to their telomerase positive counterparts in several tumor types [49], [50], [51], [52]. These observations suggest that cancer cells utilizing ALT may have compromised their vitality in exchange for the unlimited replicative potential conferred by this telomere maintenance mechanism.

### 1.2 Centrosomes, the spindle assembly checkpoint, and tumorigenesis

The centrosome is the primary microtubule organizing center in dividing mammalian cells and is composed of a pair of centrioles surrounded by a cloud of proteins that promote microtubule nucleation [53], [54]. The centrosome is duplicated in a semiconservative fashion with one daughter centriole formed next to a preexisting mother centriole, and this process only occurs once in every cell cycle [53], [55]. Centrosome amplification, the presence of greater than two centrosomes during mitosis, is a common characteristic of most solid and hematological tumors that may induce multipolar mitoses, chromosome missegregation, and subsequent genetic imbalances that promote tumorigenesis [54], [56].

Centrosome amplification may be caused by diverse mechanisms, including centrosome overduplication [53], [57], [58], [59], de novo assembly, [60], [61], [62] and mitotic failure downstream from mono- [63] or multipolar division [64], [65], [66], [67], [68], [69]. The end result of these structural abnormalities is often cytokinesis failure, which can give rise to tetraploid binucleated cells and genome instability downstream. Over time, the net result is a small population of cells that harbor the ability to manage extra centrosomes, which could account for the accumulation of cancer cells with centrosome amplification and aneuploidy.

Catastrophic aneuploidy and nonviable daughter cells are a possible tumor suppressive consequence for centrosome abnormalities [70]. However, cancer cells have developed mechanisms that overcome this fate by clustering multiple centrosomes into a “pseudobipolar” state [59], [70], [71], [72]. Cancer cells may utilize this mechanism to dampen high level aneuploidy and extreme CIN, leading to better prognostic outcomes [73], [74]. Centrosome clustering in tumor cells is not completely understood, but it is likely to rely on microtubule associated proteins and motor proteins that bundle together microtubules and centrosomes [71]. Given that centrosome clustering may be advantageous for cancer cell survival, this process may be an attractive and specific therapeutic target [71], [75], [76]. In theory, the induction of multipolarity through declustering of supernumerary centrosomes will selectively target cancer cells without affecting healthy cells [71], [75], [76], [77].

Bipolar chromosome attachment during mitosis is ensured by a quality control mechanism known as the spindle assembly checkpoint. The assembly checkpoint senses tension across kinetochores as a measure of bipolar attachment of chromosomes, and prevents the onset of anaphase in the presence of unattached and/or misattached chromosomes [78]. Any failures to sense errors will compromise the checkpoint and, potentially, induce instability.

The assembly checkpoint relies upon kinase signaling to delay cell cycle progression and correct attachment errors. Aurora kinase B, for example, detects misattached chromosomes [79], [80] and overexpression of the kinase is sufficient to disrupt the checkpoint and promote tetraploidy [81]. Moreover, mutations or expression changes in other checkpoint gene products may compromise the checkpoint and favor tumorigenesis [82], [83], [84], [85]. Lastly, oncogenic cues, such as overexpression of Aurora kinase A, may override a functioning checkpoint and enable cells to enter anaphase despite misattached chromosomes [86]. Cancer cells may take advantage of the checkpoint for their own benefit. For example, checkpoint mediated delay provides time for centrosome clustering [71], which can be manipulated by disabling or restoring assembly checkpoint function [77]. The ability to manipulate or hijack the cell's innate quality control mechanism may act as a selection pressure, and cancer cells that possess this ability may have a growth advantage over others.

Correlation between aberrant centrosome numbers and aberrant chromosome numbers dates back over 100 years [87], yet there is still a debate whether supernumerary centrosomes are the cause or the result of genomic instability, or vice versa [54]. One interesting phenomenon that may shed light on this debate is the presence of a transient tetraploid state during tumorigenesis [88].

Tetraploidy arises after cytokinesis failure following prolonged activation of the assembly checkpoint, regardless of the reason for checkpoint activation [54]. Depending on the status of tumor protein 53 (TP53), a tumor suppressor, the aborted postmitotic cells will either undergo apoptosis after prolonged cell cycle arrest or continue to cycle [56], [89], [90], [91], [92]. In *p53* null cells, a postmitotic checkpoint is compromised, which enables the cell to progress through a subsequent cell cycle with double the amount of centrosomes and genetic material [57], [89]. Consequently, each subsequent division for these tetraploid cells will be more error prone, generating more unstable and detrimental aneuploidy [88]. A TP53-dependent postmitotic checkpoint is frequently mutated during early stages of tumorigenesis [88], [90], [91], [92], which suggests that the tetraploid state serves as an intermediate for the aneuploid state observed in cancer cells [88]. In patients with Barrett's oesophagus, the presence of tetraploid cells is detected before aneuploid cells and correlates with early loss of TP53 [93]. Tetraploid cells were also isolated from *p53*^-/−^ mouse mammary epithelial cells, and these cells formed tumors in nude mice and showed increased aberrant mitoses and genomic instability in culture [94]. Therefore, regardless of how centrosome amplification or genomic instability occurs in this “chicken or egg” argument, it is clear that either event is positively enhanced by the other in promoting tumorigenesis.

### 1.3 Epigenetic mechanisms contributing to genomic instability

A plethora of studies, including more recent genome-wide profiling, have demonstrated that epigenetic changes direct different cellular phenotypes in both normal and cancer cells [95], [96], [97]. Epigenetics refer to all heritable changes that may modify gene expression without changing the primary DNA sequence, such as DNA methylation and chromatin remodelling. Epigenetic modifications are established during differentiation and are stably inherited and maintained through multiple rounds of cell division. Epigenetic processes that lead to genomic instability and ultimately malignant transformation constitute heritable changes that modulate gene expression and can also affect DNA repair dynamics [95], [96], [97].

DNA methylation consists of the addition of a methyl group at the carbon 5 position of the cytosine pyrimidine ring or the number 6 nitrogen of the adenine purine ring [98], [99]. Most cytosine methylation occurs in the context of cytosine-phosphate-guanine (CpG) dinucleotides, and occurs via a group of DNA methyltransferase enzymes resulting in silencing of gene transcription [100], [101]. Aberrant changes in DNA methylation were among the first events to be recognized in cancer [102]. Global hypomethylation in repetitive sequences of the genome can occur early during tumorigenesis and may initially predispose premalignant cells to repetitive sequence genomic instability [103]. Furthermore, hypomethylation of the promoter of oncogenes can increase their expression [104] and lead to genomic instability [105], [106]. Similarly, aberrant sequence specific hypermethylation in cancer cells can lead to further genomic instability by the silencing of genes involved in cell cycle regulation and DNA repair [107]. A prominent example is the aberrant methylation of CpG islands in the promoter regions of DNA mismatch repair (MMR) genes that result in cancer cells with a “mutator phenotype” [108], [109].

In addition to DNA methylation, histone molecules that form the primary protein component of chromatin also regulate genome stability as well as gene transcription [110]. A number of posttranslational modifications such as acetylation, deacetylation, methylation, phosphorylation and ubiquitination have been identified that alter the function of histones [111]. Various combinations of these posttranslational histone modifications have been hypothesized to form a “histone code” that dictate distinct chromatin structures that can affect genome stability pathways and transcription [95], [97], [98], [112]. Acetylation of the lysine residues at the amino (N) terminus of histone proteins removes positive charges, thereby reducing the affinity between histones and DNA to facilitate access by ribonucleic acid (RNA) polymerase and transcription factors to gene promoter regions [112]. Therefore, in most cases, histone acetylation enhances transcription while histone deacetylation represses transcription. In addition, histone acetylation can affect DNA repair by promoting histone dynamics that stimulate a DNA damage response in response to ionizing radiation [113], [114], [115], [116], [117], [118]. Similarly, histone ubiquitination can also modify DNA repair capacity [119], [120], [121], [122], [123]. Briefly, ubiquitinated histones can lead to chromatin structures that are conducive to the assembly of nucleotide excision repair complexes on damaged DNA [124], as well as both types of DSB repair pathways and cell cycle checkpoint factors critical for the DNA damage response [119], [120], [121], [122], [123], [124], [125], [126]. Monoubiquitination of histones H2A and H2B prevents chromatin compaction and facilitates assembly of the repair machinery at the damaged sites [126]. Polyubiquitination of histone H2A and H2AX is important for the retention of repair proteins, such as TP53 binding protein 1 (53BP1) and breast cancer 1 (BRCA1), at damaged loci [120], [127]. Finally, histone phosphorylation is an early event following DNA damage and required for efficient DNA repair. Upon introduction of a DSB, hundreds of histone molecules become phosphorylated within minutes at the chromatin flanking the break site, thus providing a rapid and highly amplified detection system and a focus for the accumulation of many other proteins involved in DNA repair and chromatin remodelling [128]. These examples, and numerous other observations, suggest that a vast array of epigenetic mechanisms contribute to the genomic instability in cancer cells.

### 1.4 Mitochondrial DNA alteration in human cancers

Mitochondrial genetic reprogramming and energy balance within cancer cells play a pivotal role in tumorigenesis and are duly regarded as one of the hallmarks of human cancer [129]. In 1927, Otto Warburg [130], [131] identified mitochondrial dysfunction as a key component of tumorigenesis and numerous studies have since elaborated upon the role of mitochondrial DNA (mtDNA) alterations in different human cancers [132].

Mitochondria are the key component of the oxidative phosphorylation system to generate cellular adenosine triphosphate. They uniquely possess their own DNA and generate reactive oxygen species (ROS) [132]. Most human cells contain hundreds of nearly homoplasmic (identical) copies of mtDNA, which are maternally inherited [132]. Compared to the nuclear DNA, the mutation rate of mtDNA is nearly 10 times higher and alterations are much easier to detect due to their high copy number in cancer cells.

A substantial number of studies identified somatic mtDNA mutations involving coding and noncoding mtDNA regions in various cancers [132], [133], [134], [135], [136]. Among the noncoding mtDNA mutations, a poly C mononucleotide repeat (known as D310) was frequently altered in numerous cancers and appeared to be a mutational hot spot [132]. Notably, coding mtDNA mutations targeting *respiratory complex I*, *III*, *IV* or *V* were frequent in a variety of human cancers [132], [133], [134], [135], [136], [137], [138]. Moreover, alteration of mtDNA copy number could potentially be associated with mitochondrial dysfunction leading to disease progression [132], [133]. In recent studies, a correlation between mutations in mtDNA and *epidermal growth factor receptor*, or prostate-specific antigen expression, was established in lung and prostate cancer, respectively [134], [135]. These results suggest cross talk between mitochondria and nuclear genomes maintain tumor growth.

In order to understand their role, a number of studies introduced mtDNA mutations in cancer cells. Introduction of a mitochondrial mutant *adenosine triphosphate 6* (*ATP6*) (complex V) or *cyclooxygenase 1* (*COXI*) (complex IV) increased the growth of prostate cancer cells [136], [139] or induced cancer cell proliferation and altered reactive oxygen and nitrogen species [140], respectively. In a bladder cancer study, introduction of a mutant mitochondria encoded *cytochrome B* (*CYTB*) induced bladder cancer growth and invasion, accompanied with increased ROS, lactate production and oxygen consumption [141]. Moreover, the ROS-producing *CYTB* mutant tumor cells efficiently killed normal splenic immune effector cells, which may provide tumor cells with an immune evasion mechanism [141]. In addition, mutant *CYTB* overexpression in nontumorigenic bladder epithelial cells triggered an increased mitochondrial proliferation and inhibition of apoptosis [142]. As these mutations in mtDNA were detected in human patients, the preceding studies suggest a causative role for mtDNA alterations in tumorigenesis.

## 2 Repair pathways responsible for genetic fidelity and tumor suppression

DNA is replicated with extreme fidelity in normal cells with a mutation rate of 10^−10^ per base pair per cell division. DNA damage typically occurs through the following: (1) exposure to agents such as ultraviolet irradiation, genotoxic chemicals, and ionizing radiation; (2) spontaneous DNA damaging events, such as a basic site formation; and (3) failure in normal cellular DNA processing and replication events, such as stalled replication forks. These processes induce oxidation, alkylation, crosslinking, dimerization, and strand breaks in DNA, which must be resolved. As such, repair of this DNA damage is essential to preserving genome integrity and preventing cancer.

### 2.1 Excision repair pathways

Three excision repair pathways can repair single stranded DNA damage: nucleotide excision repair (NER), base excision repair (BER), and DNA mismatch repair (MMR).

### 2.2 Nucleotide excision repair

Fidelity of genetic information transmission depends on NER, which serves to repair DNA damage caused by ultraviolet irradiation, alkylating and oxidizing agents, or chemotherapeutic drugs that form bulky, helix distorting adducts. Two sub-pathways have been identified. Global genome NER repairs damage in both strands of the DNA regardless of whether the gene is being actively transcribed [143], [144], [145]. Transcriptionally coupled NER, however, repairs transcriptionally active genes [143], [144], [145]. The two pathways are similar in that they use many of the same pathways, but global genome NER uses xeroderma pigmentosum complementation group C (XPC)-RAD23 homolog B (HR23B) and DNA damage binding protein 1 (DDB1)-DDB2/XPE proteins to recognize distortions in the double helix while transcriptionally coupled NER occurs at regions where RNA Polymerase II has stalled [146], [147], [148], [149], [150]. Genetic polymorphisms of NER gene products associate with human diseases, including xeroderma pigmentosum, which can lead to severe cases of skin cancer.

### 2.3 Base excision repair

The BER pathway fixes damaged DNA bases (reviewed in [151]). These lesions are recognized and removed by specific DNA glycosylases, which cleave the glycosidic bond between the damaged base and the sugar of the DNA backbone. In more complex lesions, proliferating cell nuclear antigen (PCNA), flap endonuclease 1 (FEN1), and DNA polymerase (POL) β, with or without POLδ/ɛ, act to repair the lesion. This complex set of events in BER is facilitated by poly (ADP-ribose) polymerase 1 (PARP1), which recruits proteins involved in the DNA repair response, such as X-ray repair cross-complementing protein (XRCC)1, DNA ligase, and DNA polymerase [152], [153].

Because cells are constantly subjected to DNA damaging conditions, the BER pathway is crucial to preserving genome integrity. This is exemplified by the embryonic lethality of mice that possess knockouts of key components of this pathway [154], [155], [156]. A biallelic germline defect within a DNA glycosylase, *mutY Homology (MUTYH)*, was initially found in families that had excess colorectal tumors with *somatic* mutations in the adenomatous polyposis coli gene [157]. A subsequent larger study revealed that biallelic germline *MUTYH* defects conferred 93 fold excess risk of colon cancer with penetrance by age 60 [158], [159] and may also confer increased risk for endometrial cancer [160]. Mutations in another glycosylase, *8 Oxoguanine (OGG1)*, have been associated with laryngeal cancers [161] while gastric cancers harbor inactivating mutations in *glycosylase nei endonuclease VIII-like 1 (NEIL1)* [162]. Taken together, these studies confirm the importance of BER in the suppression of carcinogenesis.

### 2.4 DNA mismatch repair

Some evidence suggests that proofreading activity of replicative DNA polymerases and MMR machinery act in series in mammalian cells [163], [164], [165], [166]. MMR targets could generally be classified into base/base mispairs and large insertion–deletion loops. At the forefront of error recognition, MutS protein homolog (Msh) 2 pairs with Msh6 or Msh3, to form MutSα (Msh2/Msh6) and MutSβ (Msh2/Msh3). Whereas the former is mostly responsible for base/base mispairs, the latter targets large insertion–deletion loops. To initiate the repair process, MutL homolog 1(Mlh1)/Pms2 heterodimers (MutL homologues), in the presence of exonuclease1, interact with MutS complexes to create nicks in the 3′ and 5′ of the nascent strand containing the mismatch. Following nick creation, enzymes required to repair the damage site are recruited and resynthesis of the DNA is carried out by POLδ/ɛ. As a nexus for DNA damage sensing and cell death, MMR machinery play an important role in recognizing damaged DNA and relaying signals downstream to activate a G2/M cell cycle checkpoint.

### 2.5 Double strand break repair and cancer predisposition

The DSB is the most lethal form of DNA damage, as it can lead to significant DNA damage by multiple genomic changes, including translocation formation, deletions, and amplifications, resulting in heritable cellular genomic instability/damage that can lead to malignancy [167], [168], [169]. DSBs are repaired by both HR and NHEJ repair pathways. NHEJ repair occurs throughout the cell cycle, while HR prevails in S and G2 phase cells (reviewed, [167], [168], [169]). NHEJ repair joins broken DNA ends without identifying DNA sequence homology and is therefore highly error prone [169]. HR repair is dependent upon DNA sequence homology and therefore is relatively error free [168], [169], [170].

Errors in the NHEJ pathway may generate inappropriate dicentric chromosomes that are covalently joined (reviewed, [171], [172]). These dicentric chromosomes may break during anaphase, producing new dicentric chromosomes through further NHEJ [171], [172]. This process is known as the breakage fusion bridge (BFB) cycle, which is important in telomere related genome instability [172]. Damaged telomeres will be processed by NHEJ, unless the broken ends are healed by a new telomere, and continuation of the BFB cycle can result in complex chromosomal rearrangements that include gene loss, gene amplification and unbalanced translocations [171], [172]. BFB cycles are self-perpetuating and result in genetic heterogeneity in a variety of cancers [173].

HR repair involves multiple gene products some of which are involved in repair of stalled replication forks [170]. The DSB is recognized by a Mre11-Rad50-NBS1 complex which recruits many different proteins, including proteins with topoisomerase, endonuclease, and helicase activity. Eventually a “synaptic complex” is formed which allows homologous single strand DNA (ssDNA) to invade and anneal to complementary DNA. DNA polymerase then fills in the ssDNA gap and the synaptic complex is resolved. Both crossover and non-crossover products can be created by this process [167], [168], [169], [170]. Interestingly, loss or mutation of many of these gene products is associated with specific cancer prone diseases.

BRCA1 and BRCA2 are key players in the HR repair pathway and act as tumor suppressors by maintaining genome stability. Linkage studies in families with early onset breast cancer detected the presence of a breast cancer susceptibility gene, BRCA1 [174]. Subsequently, mutations in *BRCA1* were confirmed in families with early onset breast and ovarian cancer [175], [176], [177], [178]. Later, a novel locus, encoding *BRCA2*, was discovered and linked to breast cancer susceptibility [179], [180], [181]. In one meta-analysis, cumulative breast and ovarian cancer risks for *BRCA1* mutation carriers are 57 and 40%, respectively, while, for carriers of *BRCA2* mutations, these risks are 49 and 18%, respectively [182]. Furthermore, hereditary *BRCA* mutations have been also linked to pancreatic, prostate, and colon cancers [183]. Of interest, germline mutations in *BRCA1* versus *BRCA2* associate with different subtypes of breast cancer. BRCA1 associated cancers are of the more aggressive triple negative subtype and appear at an earlier age than sporadic tumors. In contrast, BRCA2-associated tumors relate mostly with hormone receptor positive breast cancers.

Like BRCA1 and BRCA2, partner and localizer of BRCA1 (PALB2) promotes genome integrity through its role in DSB repair. It binds and colocalizes with BRCA2 in the nucleus to stabilize BRCA2 foci and facilitate the intra S phase checkpoint and HR repair [184]. Germline mutations in *PALB2* confer a 2–5 fold increase in breast cancer risk [185], [186] and germline mutations have been recently found in African American breast cancer patients [187], [188]. *PALB2* mutations have also been observed in 1% of Chinese women with early onset breast cancer [189]. Interestingly, exome sequencing identified *PALB2* as a pancreatic cancer susceptibility gene [190] and *PALB2* mutations have been found in patients with familial pancreatic cancers [191], [192].

Fanconi anemia (FA) is an autosomal recessive disorder characterized by congenital defects, CIN, hypersensitivity to DNA crosslinks, bone marrow failure, and predisposition to cancer [193], [194]. Fifteen FA or FA-like genes have been identified, all of which are involved in coordinating DNA repair through the FA/BRCA pathway. Interestingly, two of these genes are *BRCA2 (FANCD1)* and *PALB2 (FANCN)*, thus revealing interplay between FA and HR [195], [196]. Patients with FA have increased susceptibility to breast, ovarian, and oral cancers. Additionally, heterozygote carriers of germline mutations in FA genes may also harbor an increased risk to develop cancer. Importantly, unlike the BRCA-associated cancers, tumors from FA patients, or with acquired FA defects, may be hypersensitive to crosslinking agents such as cisplatin and mitomycin C, and also are hypersensitive to radiation [197], [198], [199]. Finally, biallelic loss of *ataxia telangiectasia mutated* (ATM) results in ataxia telangiectasia, a disease characterized by a roughly 1000 fold increased lymphoma incidence [200], [201].

## 3 Therapeutic targeting of genomic instability

**Fig. 1:**
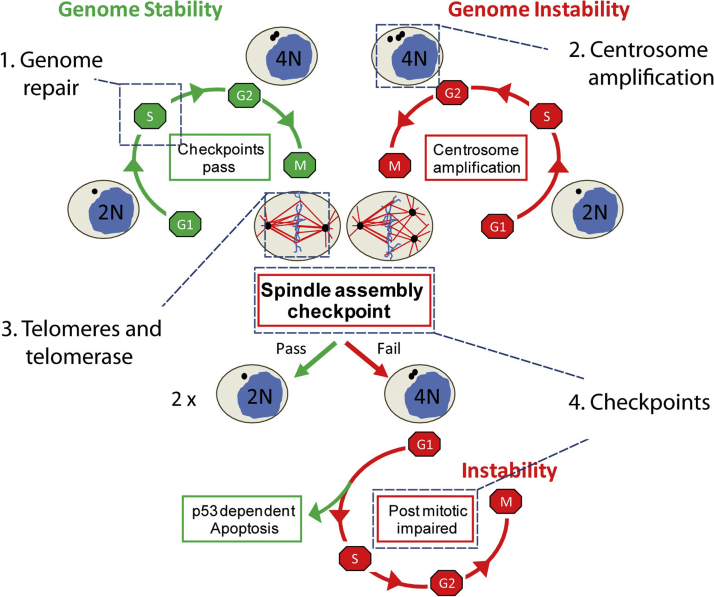
Genome stability is dependent on faithful DNA repair and chromosome segregation during cell division. During S phase, the centrosome and genomic material are replicated concurrently, and replication errors are repaired prior to mitotic entry (1). During mitosis, equal segregation of chromosomes requires a bipolar mitotic spindle, telomere preservation and the completion of the spindle assembly checkpoint. Ectopic amplification of centrosomes (2), telomerase dysfunction (3) and failure of the spindle assembly checkpoint (4) may result in aborted mitosis. Mitotic failure gives rise to a single tetraploid cell (4 N) instead of two diploid cells (2 N). This tetraploid cell can progress through the cell cycle should the TP53-dependent post mitotic checkpoint fail to induce apoptosis or senescence (4). Thus, genomic instability is propagated in subsequent cell cycles.

Current standard therapies for cancer often involve agents or strategies that damage the DNA, which can also damage noncancerous tissues. New treatments that target genomic instability (Fig. 1) may minimize these off target toxicities to normal tissues.

### 3.1 Targeting DNA repair pathways in cancer therapy

Drugs that target DNA repair proteins have shown preclinical and/or clinical efficacy in potentiating DNA damage (reviewed in [202]). Synthetic lethality, whereby deficiencies in parallel pathways are only cytotoxic when both pathways are defective, is a novel strategy that may selectively target cancerous cells defective in DNA repair [203]. Synthetic lethality was illustrated in BRCA deficient cells, which exhibited profound sensitivity to inhibition of PARP [204], [205]. This was due to conversion of unrepaired single strand breaks into DSBs during DNA replication due to the BRCA deficiency. Persistence of the unrepaired DSBs led to profound cellular cytotoxicity. The potential efficacy of PARP inhibitors in patients with BRCA-associated cancers has been reported in multiple clinical trials [206], [207], [208], [209], [210], [211]. Perhaps more important is the high therapeutic index of these compounds, since noncancerous cells in this patient population still maintain one wild type allele and thus remain HR proficient. Natural products have also been shown to act on PARP, such as isothiocyanates, which are found in *Brassica* vegetables. Isothiocyanates stimulate the proteolytic cleavage of PARP [212]. Recent work implicates vitamin D as a possible treatment mechanism to supplement PARP treatment [213]. BRCA1-deficient cells bypass growth arrest by activating cathepsin L (CTSL)-mediated degradation of 53BP1. Vitamin D depletes or inhibits CTSL leading to increased genomic instability and compromised cancer cell proliferation after irradiation and treatment with PARP inhibitors [213]. Selenocysteine can induce ROS formation, which can lead to DSBs in cancer cells but not in normal human fibroblasts [214]. Thus, DSB repair deficient cancers may be sensitive to treatment with selenium compounds.

In addition to defective DSB repair, inappropriate HR often results in a significant predisposition to cancer development [200], [201]. Many human malignancies with HR deficiency show increased sensitivity to chemotherapy agents that cause DSBs, such as ionizing radiation, bleomycin, and cisplatin. Additionally, agents which inhibit DNA replication, such as bifunctional alkylating agents and topoisomerase inhibitors, also preferentially inhibit the growth of HR deficient malignant cells [170], [215], [216]. There has been intense interest in identifying HR deficiencies in human tumors and targeting these tumors with DSB-inducing chemotherapeutic agents. Since ∼25% of human malignancies show HR defects [170], targeted treatment could eventually play a significant role in chemotherapy. As tumors often overexpress specific proteins involved in HR, this approach might also preferentially target tumor over normal tissue [170], [215], [216], [217]. A major challenge in this area is the efficient and accurate identification of HR deficiency in human malignancies.

Some of the proteins that have been proposed as useful targets to inhibit HR include ATM, checkpoint kinase (CHK)1&2, ataxia telangiectasia and Rad3 related (ATR), and the FA pathway proteins [170]. Many inhibitors that target these enzymes are either in preclinical development or in the early phases of clinical development [218], [219]. Resveratrol may activate sirtuin 1 (Sirt1) activity [220], which is a nicotinamide adenine dinucleotide-dependent deacetylase that is known to activate DNA repair. Studies in mice have shown that *Sirt1*^+/−^; *p53*^+/−^ mice develop tumors in many different tissue types but mice treated with resveratrol display a reduced amount of tumorigenesis [221], indicating that resveratrol could act to prevent and/or treat cancers in patients that have reduced Sirt1 function. Although in early development, therapies that specifically alter HR are a promising area of research and may contribute to targeted chemotherapy regimens that are more personalized and effective.

### 3.2 Targeting microsatellite instability

MMR inactivation is associated with the lack of repair of replication errors leading to an increase in spontaneous mutation rate [222]. A marker of defective MMR is microsatellite instability (MSI), or numerous alterations in the lengths of microsatellites [223], [224]. Tumors displaying MSI are said to exhibit a “mutator phenotype”, with a dramatic predisposition to somatic mutations.

The critical role of MMR pathways in tumorigenesis is exemplified by the fact that germline mutations in the genes involved in MMR predispose to cancer development [225]. In the case of colorectal cancer (CRC), MMR deficiency is estimated to be present in 15 to 17% of all primary cancers, including both sporadic CRC and Lynch syndrome (then called hereditary nonpolyposis colorectal cancer), though through different mechanisms [223], [226], [227]. Lynch syndrome is characterized by inactivating germline mutations to *MSH2, MSH6, PMS2, or MLH1*, whereas *MLH1* expression is silenced due to biallelic hypermethylation in sporadic CRC [228], [229], [230], [231], [232]. *MLH1* methylation results from extensive aberrant promoter methylation [233], [234]. The 3′ end of the *MLH1* promoter, proximal to the start codon, is most commonly methylated [227]. Methylation of the 5′ end of the *MLH1* promoter can also occur, however, the methylation pattern must extends to the 3′ end to be deleterious [227]. Loss of *MLH1* expression increases with age and protein expression is lost by ∼50% in patients who are 90 years or older [235]. The exact mechanism(s) behind *MLH1* silencing remain unknown, but may result from abnormal methylation [236], structural chromatin changes that increase accessibility to promoter regions [237], or genomic damage [238]. Tumors with mutations in the *MLH1* gene are rare, which suggests that hypermethylation of the *MLH1* promoter is an important event in neoplastic transformation in sporadic CRC [234].

The extent of MSI in CRC has been classified as MSS (microsatellite–stable), MSI-H (with high level of instability), and MSI-L (with low level of instability) [239]. Classification between MSI-H and MSI-L depends on which MSI markers are present and their proportions [240]. These markers include mononucleotide repeats, such as BAT25, BAT26, and BAT40, and the dinucleotide repeats D5S346, D2S123, and D17S250 [241], [242], [243], [244]. Dinucleotide markers are present in both MSI-H and MSI-L cancers, whereas mononucleotide markers are specific for MSI-H cancers [241]. In MSI-H tumors, more than 30% of these markers are unstable, while in MSH-L tumors, 10–40% of these markers are unstable, and MSS tumors demonstrate no unstable markers [241], [245], [246], [247], [248]. MSI-L and MSS tumors are frequently grouped together due to similarities in their clinical features and gross abnormalities [248]. MSI-H is most prevalent in sporadic CRC, observed in 10–15% of all cases [240], [248].

The predictive value of MMR status as a marker of response to 5 fluorouracil, irinotecan and other drugs is still controversial [249]. Recently, two large retrospective analyses from several randomized trials confirmed the detrimental effect of a 5 fluorouracil-based adjuvant therapy in stage II colorectal patients [250], not applicable to stage III patients [251]. These latter authors, however, reported that MSI stage III tumors harboring genetic mutation in the MMR genes seem to benefit from the 5 fluorouracil adjuvant therapy. These data imply that molecular differences within the MSI subgroup influence the response to 5 fluorouracil.

The CRC MSI subgroup represents a cancer with a defined molecular etiology, a characterized mutational profile and an established genotype–phenotype relationship, which may enable synthetic lethal approaches that target MMR deficiency. High throughput experiments revealed synthetic lethal interactions between *MSH2* and *POLB*, between *MLH1* and *Polymerase G* gene [252], between *RAD54B* and *FEN1* [253], [254], between *MLH1/MSH2* and *PTEN-induced putative kinase 1* gene [255], and the preferential effect of methotrexate in MMR deficient systems [256]. These synthetic interactions may induce or accumulate ROS [257], [258]. A phase II randomized clinical trial in MLH2-deficient metastatic CRC (NTC00952016) is currently underway [256]. Combination therapy with methotrexate and PARP inhibitors may be effective against tumors with MMR mutations. Methotrexate elevates ROS and DSBs and the combination of MMR mutation and PARP inhibition may attenuate repair and induce growth arrest or apoptosis [259], [260], [261].

### 3.3 Targeting gene expression of cell cycle and DNA repair components

RNA interference (RNAi) may enable personalized antitumor therapies. A number of RNAi-based studies have silenced genes responsible for tumor cell growth, metastasis, angiogenesis, and chemoresistance [262]. For example, siRNA targeting of Cyclin E suppressed tumor development [263]. Epigenetic regulation of gene expression is an alternative approach. Resveratrol, a phytoalexin produced by plants such as the Japanese knotweed, prevents hypermethylation of the BRCA1 promoter [264], and may be effective for triple negative or basal subtype breast cancers. Other natural compounds, like genistein and lycopene, can alter DNA methylation of the *glutathione S transferase p1* (*GSTP1*) tumor suppressor gene [265].

### 3.4 Targeting centrosome abnormalities

Centrosome amplification is an important process during early stages of cancer development (see Section 1.2). Though the mechanism(s) behind centrosome amplification remain elusive, TP53 negatively regulates centrosome amplification through a TP53-p21-CDK2 signaling loop [266], [267]. Moreover, TP53 induces apoptosis through transactivation of proapoptotic genes and transrepression of antiapoptotic genes [268]. Thus, TP53 provides an interesting link between two major cancer processes, centrosome amplification and apoptosis dysregulation [268]. In one study, the loss of TP53, or treatment with 5 fluorouracil, promoted centrosome amplification in HCT116 cells and those cells with supernumerary centrosomes were more acutely sensitive to resveratrol [268]. However, TP53 defective cancer cells that resist 5 fluorouracil treatment are prone to centrosome amplification and downstream genome instability [269]. The presence of supernumerary centrosome can also be problematic for cancer cells. Clustering excess centrosomes may be a necessary prosurvival pathway for cancer cells and thus an attractive target [70]. Griseofulvin, an antifungal drug that suppresses proliferation in tumor cells without affecting non-transformed cells, declusters centrosome, although the precise mechanisms behind the drug's action remain unknown [71]. In a similar fashion, depletion of a kinesin-like motor protein can selectively kill tumor cells with supernumerary centrosomes [77]. Finally, the PARP inhibitor PJ34 also declusters supernumerary centrosomes without deleterious effects on spindle morphology, centrosome integrity, mitosis, or cell viability in normal cells [270].

## 4 Prevention of genomic instability and human cancer

There is no question that optimizing nutrient intake plays a significant role in stabilizing the genome. In recent years, an increasing number of biomarkers of genome integrity, including telomere length and mtDNA deletions, have been utilized in establishing recommended daily intakes for nutrients [271]. In several cases, such an approach has led to substantial changes in the levels of various nutrients that populations had been previously advised to consume. These findings highlight the need to better optimize an individual's diet to their personal genetic makeup, which in turn has prompted the emergence of nutrigenomics, a new field that aims to determine how a particular genotype or expression profile correlates to nutrient metabolism, absorption, etc. (reviewed in [272], [273]).

### 4.1 Vitamins–carotenoids

Since Peto et al. [274] concluded that the evidence pointed to a cancer preventive role for β carotene, many placebo-controlled carotenoid intervention trials have been carried out with disease and mortality as outcomes. Early findings were that, in subjects who were smokers and/or asbestos workers, there was a significant increase in lung cancer incidence [275]. A recent meta-analysis confirmed that a significant increase in mortality is associated with vitamin A, β carotene or vitamin E supplements [276]. When determining the effects that dietary supplements and other compounds have on cancer prevention, it is important to take into account the different types of data: conventional intervention studies, animal experiments, cell culture studies, or human intervention trials based on biomarkers. This is important because of the nature of each type of experiment and the information that can be obtained from them.

#### 4.1.1 Human biomarker trials with molecular endpoints

Human trials with carotenoids [277], [278], [279], [280], [281], [282], [283], [284], [285], [286] were mostly cross sectional or case control studies, with a few intervention trials. In general, a negative correlation was seen between blood carotenoid levels and various biomarkers of DNA damage, and intervention trials tended to show a decrease in DNA damage or no effect.

#### 4.1.2 Animal experiments

Most animal experiments [287], [288], [289], [290], [291], [292], [293], [294], [295], [296], [297], [298], [299], [300] involved treatment of rats or ferrets with genotoxic agents during or after carotenoid supplementation, and generally decreased levels of DNA damage were reported. Another study [301] looked at base oxidation in leukocytes and oxidation products in urine, reporting a carotenoid-induced decrease in the former (but no effect on urinary biomarkers). There was a decrease in endogenous DNA oxidation in liver of mice fed tomato paste (rich in lycopene) [302].

#### 4.1.3 Cell culture experiments

We found eleven cell culture studies [287], [303], [304], [305], [306], [307], [308], [309], [310], [311], [312] with provitamin A carotenoids (α and β carotene, β cryptoxanthin, retinoic acid, retinal and retinol), and eight with nonvitamin A carotenoids (lycopene, lutein, astaxanthin or zeaxanthin) [294], [304], [313], [314], [315], [316], [317], [318]. Experiments in most cases involved cotreatment with DNA damaging agent and carotenoid. Concentrations of carotenoid varied widely, from less than 1–100 μM. Here we found a very clear pattern in the results, depending on the type of carotenoid and the concentration: while the non-vitamin A carotenoids invariably resulted in a decrease in DNA damage, the provitamin A carotenoids at low concentrations either had no effect or decreased DNA damage, while at concentrations above about 5 μM, increases in damage were the norm. Potential prooxidant effects of carotenoids can probably be ruled out as a cause of this DNA damage, since there is no obvious reason why provitamin A carotenoids should be more likely to act as prooxidants. Instead, we should perhaps be looking at downstream effects of vitamin A itself, on transcription, via retinoic acid and retinoic acid receptors binding to retinoic acid response elements present in the regulatory sequence of many genes.

In a review of effects of carotenoids on DNA repair [319], we found relatively few reports. Cells from the spleen of rats supplemented with carotenoids (plus nicotinamide and zinc) showed accelerated repair of DSBs induced by radiation, and lymphocytes from human subjects given the same supplement mix were faster at rejoining hydrogen peroxide induced breaks [184]. Mixed carotenes plus α tocopherol as a supplement in humans had no effect on DNA repair [320]. DSB rejoining was faster in Molt 17 cells in the presence of β carotene, lutein or β cryptoxanthin [321], and in HeLa and Caco2 cells with β cryptoxanthin [306]; but no effect was seen in lymphocytes incubated with β carotene or lycopene [322] or with vitamin A [311].

Lung cells from ferrets supplemented with β carotene were tested for BER capacity with an *in vitro* comet-based assay, and showed an increase in activity [323]. B cryptoxanthin enhanced BER of 8 oxoguanine in HeLa and Caco2 cells [306], but no effect of carotenoids was seen in Molt 17 cells [321] or lymphocytes [320].

The pattern that emerges from this survey of carotenoid effects on DNA damage is that, in cell culture at least, while nonvitamin A carotenoids tend to decrease damage (whether endogenous or induced), at whatever concentration, the provitamin A carotenoids show a clear tendency to cause or increase damage at high concentrations. Whether this can account for the apparent harmful effects of β carotene as seen in the human clinical trials is not possible to answer at present.

Glutathione is another important antioxidant that can improve outcomes for patients with cancer and can help reduce treatment toxicity. Some studies have shown that supplementation with glutathione can reduce the toxicity of chemotherapy agents such as cisplatin and cyclophosphamide during treatment [324], [325]. Interestingly, while the antioxidant properties of glutathione may reduce treatment toxicity, the same properties can make tumor cells resistant to chemotherapy when glutathione is present in high levels in the cells [326], [327]. A *GSTP1* polymorphism (*GSTP1* Ile105Val), which has a seven fold higher efficiency, has been linked to a reduced survival rate in cancer patients further emphasizing that while glutathione may be able to reduce treatment toxicity, it can potentially also confer an advantage to the tumor cells as well [328], [329].

An additional aspect that may lead to conflicting results regarding the efficacy of antioxidants in cancer treatment is the oxidative stress that tumor cells experience in their microenvironment. Tumor cells have been shown to undergo the Warburg effect, which is where they produce energy primarily through glycolysis rather than through aerobic respiration ([131]; reviewed in [330]). The production of lactate in tumor cells rather than pyruvate, an antioxidant, increases the load of ROS and increases oxidative stress in the cancer cells. Recent work has suggested that cancer cells might be targeted by using 3 bromopyruvate, an inhibitor of the glycolysis enzyme hexokinase II, to amplify the Warburg effect in cells [331].

### 4.2 Other vitamins

A range of B vitamins, including niacin (vitamin B3), folate (vitamin B9), and vitamin B12, significantly interact to maintain the stability of both nuclear and mitochondrial genomes. For example, a niacin deficiency, common in certain populations, impairs the function of the PARP family of enzymes, identified above as critical to DNA repair. A folate deficiency, especially in the presence of suboptimal levels of vitamin B6 and vitamin B12, may have significant effects on the expression of chromosomal fragile sites, leading to chromosome breaks, micronuclei and deletions of mtDNA. It may also lead to reduced telomere length. There are considerable interindividual differences in people's capacity to absorb and metabolize these vitamins, dependent upon genotype and epigenotype [332].

Vitamin C is considered an antioxidant, and is present at high concentrations (mM) in certain tissues such as the eye. Effects on various markers of genome stability were shown to depend on individual diet-derived vitamin C concentrations, and also on exposure to xenobiotics or oxidative stress [333]. Vitamin D is also critical in the maintenance of genome stability, possibly through protection against oxidative stress, chromosomal aberrations, telomere shortening and inhibition of telomerase activity [334].

### 4.3 Minerals

While a number of minerals are typically considered as toxicants, there are several that are essential micronutrients, albeit usually with a narrow window of efficacy as compared with toxicity. These include iron [335], selenium [336], and zinc [337].

Selenium provides a useful illustration of the complexities of reaching agreement on optimal population levels. The population as a whole shows a “U” shaped curve for functionality, where low and high selenium levels both increase genomic instability. Optimal levels of selenium may protect against DNA or chromosome breakage, chromosome gain or loss, damage to mtDNA, and detrimental effects on telomere length and function. One example of how selenium can function is by protecting genome stability through a BRCA1-dependent mechanism [338]. When cells are supplemented with selenium, there is reduced DNA breakage and the number of aneuploid cells is reduced when compared to control cells. Unfortunately, these optima differ among individuals and according to the form of selenium in the diet [336], [339]. Various genetic polymorphisms have been shown to affect the uptake and utilization of selenium among individuals.

### 4.4 Other dietary factors

Diets high in plant-based foods have been associated with decreased cancer risks [340]. Lim and Song [341] discuss how certain dietary components, common in plant foods, can alter DNA methylation levels, affecting genome stability and transcription of tumor suppressors and oncogenes. Much of the available data exist for folate, since this is a well-recognized nutritional factor in one-carbon metabolism, acting to supply the methyl units for DNA methylation. This has been shown to be especially important in the maternal diet as a lack of folate can lead to hypomethylation of some genes in their offspring. One well studied example of this is the Agouti mutation in mice, which affects coat color, as well as making the offspring more susceptible to cancer and obesity [342], [343]. The Agouti mutation has been shown to be a lack of methylation at the promoter of the Agouti gene [344]. Pregnant mice that were fed bisphenol A had offspring that exhibited hypomethylation of the Agouti gene but, by feeding them dietary supplements of folate, methylation status was rescued [345]. This data demonstrates the importance of the maternal diet during development to outcomes even later in life for their offspring. In other systems, folate supplements during pregnancy have also been shown to be protective against neuroectodermal brain tumors [346].

Alcohol, various polyphenols, phytoestrogens and lycopene also have demonstrable effects. Indeed, there is compelling evidence that a considerable range of plant polyphenols may stabilize genomic DNA, through various processes, including effects on DNA methylation [347]. Duthie [340] suggested that the evidence is particularly strong for berry phytochemicals, specifically anthocyanins (a class of flavonoids), which modulate various biomarkers of DNA damage and carcinogenesis, in both *in vitro* and *in vivo* animal studies. However, evidence for cancer preventive effects in human studies is currently weak.

Accumulating evidence shows that genome integrity is highly sensitive to nutrient status, and that optimal levels may differ among individuals. Many investigations to date are limited by considering only the effects of single nutrients, without looking at the potential interactions among these, and of nutrients with toxicants in the diet. Many currently available studies also suffer from a failure to consider the effects of genetic susceptibility. In subsequent work, it will be critical to consider modifying and interactive effects with deficiencies in nutrients required for effective DNA damage response, and DNA repair.

Hyperglycemia and a high fat diet have been shown to be positively correlated with an increased risk of cancers, such as breast and endometrial cancers. Hyperglycemic diets have been shown to increase the levels of many signaling molecules [348]. Rats that were fed high fat diets had an increased risk of breast cancer in their progeny [349]. These results were similar to those seen when mice are treated with estradiol. In addition to an increased risk of developing cancer, hyperglycemia, diabetes, and obesity have been linked to a worse prognosis. Advanced breast cancer patients with high blood glucose levels had a lower rate of survival than those with normal sugar levels [350] and obese adolescents with pediatric acute lymphoblastic leukemia had a higher likelihood of relapse than a normal cohort [351].

## 5 Complementary effects on the enabling characteristics of cancer while targeting genomic instability

Treatments that are less cytotoxic but can also act on multiple different cancers and pathways that contribute to cancer formation is an important goal (Table 1). Work focusing on the effects of vitamin D, vitamin B, selenium, carotenoids, PARP inhibitors, resveratrol, and isothiocyanates has shown promising results (Table 2).

During cancer formation, genetic instability interacts with many other pathways that are integral to the survival and proliferation of the cancer cells, such as inflammation, immune system evasion, or apoptosis resistance. Preventing and/or treating genomic instability can cause tumor cells to lose: (1) their replicative immortality; (2) their ability to evade the immune system; and/or, (3) their ability to evade antigrowth signaling (Table 1). For example, in MSI-H CRCs, the immune response can be evaded by mutations in the neoantigens caused by defects in DNA repair machinery [352]. In this case, by preventing genomic instability, it could be possible to minimize the mutations that lead to immune system evasion.

Sustained proliferative signaling is required for cancer cell growth and vitamin D and resveratrol are able to inhibit this signaling [353], [354], [355]. There are no known interactions for the other compounds, except selenium, which inhibits growth in some cases while inducing it in others [356], [357]. In a related characteristic of cancer cells, evasion of antigrowth signaling, all compounds are able to inhibit growth except vitamin B, which shows no relationship, and carotenoids, which have mixed results [255], [358], [359], [360], [361], [362], [363], [364], [365], [366]. Similarly, all of the compounds are able to prevent replicative immortality by impairing telomerase activity or inducing senescence [364], [367], [368], [369], [370], [371], [372], [373], [374], [375], [376] and increasing cell death except for vitamin B, which has no known relationship to apoptosis [377], [378], [379], [380], [381], [382]. Dysregulated metabolism also contributes to cancer cell growth and all of the compounds had complementary effects on metabolic pathways except vitamin D and PARP inhibitors, which have no known link [268], [338], [364], [383], [384], [385], [386].

Cancer cells use inflammatory agents in the microenvironment to promote their proliferation and survival. One important inflammatory signaling molecule is nuclear factor kappa-light-chain-enhancer of activated B cells (NFκB), a transcription factor whose aberrant regulation has been linked to cancer [387], [388]. Inflammatory signaling, including that of NFκB, can be affected by the diet. It has been shown that compounds found in cruciferous vegetables can reduce NFκB signaling in pancreatic cancer cells [389]. Polyphenols have also been shown to suppress transcription factors upstream of NFκB [390]. Inflammation, in general, is also inhibited by all of the compounds, except vitamin B [354], [391], [392], [393], [394], [395], [396], [397], [398], [399], [400], [401], [402], [403], [404], while only vitamin D, carotenoids, and resveratrol prevent the tumor cells from evading the immune system [405], [406], [407].

Tumors need specialized environments to grow and thrive in. As the tumor grows, new blood vessels need to form to provide the cells with oxygen and all of the treatment options selected are able to inhibit angiogenesis, except vitamin B and selenium, both of which show mixed results [408], [409], [410], [411], [412], [413], [414], [415], [416], [417], [418], [419]. Interestingly though, in regards to other factors that contribute to the tumor microenvironment, all compounds are able to provide therapeutic value [395], [420], [421], [422], [423], [424].

Effective treatments to prevent tissue invasion and metastasis are important as these stages of cancer are associated with poor outcomes. It has been found that all of the targeted treatments are able to inhibit/prevent these pathways except for resveratrol and PARP inhibitors, which have no known relationship [363], [420], [421], [425], [426], [427], [428], [429], [430], [431]. Further work and clinical trials will have to be performed to understand the full benefit of these compounds in regards to cancer treatment.

## 6 Conclusion

Genomic instability plays a critical role in cancer initiation and progression. The fidelity of the genome is protected at every stage of the cell cycle. In cancer, the presence of aneuploid or tetraploid cells indicates the failure of one or many of these safety nets. The resultant genomic heterogeneity may offer the cancer “tissue” a selection advantage against standard of care and emerging therapies. Understanding these safety nets, and how they are bypassed in cancer cells, may highlight new and more specific mechanisms for cancer prevention or therapeutic attack.

The therapeutic targeting of genomic instability may dampen other enabling characteristic of tumors cells, such as replicative immortality, evasion of antigrowth signaling, and tumor promoting inflammation. To this end, vitamins, minerals, and antioxidants, such as vitamin D, vitamin B, selenium, and carotenoids, as well as nutraceuticals, such as resveratrol, have shown remarkable plasticity in elucidating antitumor responses. In addition to alleviating genomic instability, these compounds are known to inhibit proliferative signaling [353], [354], [355], attenuate oncogenic metabolism [268], [338], [364], [383], [384], [385], [386], and block inflammation [354], [391], [392], [393], [394], [395], [396], [397], [398], [399], [400], [401], [402], [403], [404]. However, caution must be applied as certain antioxidants, such β carotene, may promote carcinogenic processes in a dose- and context-dependent manner.

While mortality rates associated with heart disease and stroke have been reduced ∼70% in the last 50 years, mortality rates associated with cancer remain largely unchanged. This is likely due to our ability to manage the risk factors for heart disease and stroke and our inability to detect and prevent genomic instability and cancer. However, diet and lifestyle are two of our great hopes in this area. In particular, antioxidants are critical for the prevention of DNA damage that enables cancer initiation and growth. Growing evidence shows that vitamins, minerals, and other dietary factors have profound and protective effects against cancer cells, whether they are grown in the lab, in animals, or studied in human populations. A better understanding of the effects and synergy of these dietary factors in the prevention and treatment of genomic instability is critical to the future reduction of mortality associated with cancer.

## Author's contributions

The following authors composed the indicated sections: HC, CAM, AKM (Mechanisms underlying genomic instability), PTT (epigenetic mechanisms), SD and DS (Mitochondrial genetics), GD, ESY, SR (DNA repair pathways), ARC and LRF (cancer prevention), SP and MM (targeting with RNAi). AA, AA, SSA, KA, ASA, DB, AB, CSB, SC, MC, MRC, HJ, GG, DH, BH, WNK, SM, EN, XY and KH (cross validation in Table 1, Table 2). LRF, MC, and CAM integrated and edited the sections.

## Conflict of interest statement

The following authors declare that there are no conflicts of interest: LRF, HC, ARC, MC, GD, SD, MM, AKM, AA, AA, SSA, KA, ASA, DB, AB, CSB, SC, MRC, HF, GG, DH, WGH, WNK, SIM, EN, XY, KH, VRP, PR, SR, RS, DS, PTT, and CAM. ESY has material transfer agreements with AbbVie, Eli Lilly, Bristol Myers Squibb, and Cerion NRx.

## Figures and Tables

**Table 1:** Cross-validation for priority targets against genomic instability.

Other cancer hallmarks	Priority targets for genomic instability				
	Prevent DNA damage	Enhance DNA repair	Target deficient DNA repair	Block centrosome clustering	Inhibit telomerase
Sustained proliferative signaling	0	0	0	0	+ [432], [433]
Tumor-promoting inflammation	− [434], [435], [436]	− [434], [435], [436]	− [434], [435], [436]	+ [437], [438]	+ [439], [440]
Evasion of anti-growth signaling	+ [441], [442]	+ [441], [442], [443]	+ [444]	+ [445], [446]	+ [447]
Resistance to apoptosis	± [448]	± [448]	± [448]	0	+ [449]
Replicative immortality	+ [450], [451]	0	0	+ [452]	n/a – same target
Dysregulated metabolism	± [453]	± [453]	± [453]	+ [454]	+ [455], [456]
Immune system invasion	+ [352]	+ [352]	+ [352]	0	0
Angiogenesis	− [457], [458], [459], [460], [461]	− [457], [458], [459], [460], [461]	0	+ [462]	+ [463], [464], [465]
Tissue invasion and metastasis	+ [466], [467]	+ [466], [467]	+ [466], [467]	+ [468], [469]	+ [470], [471], [472], [473], [474]
Tumor microenvironment	+	+ [475]	+ [476]	+ [437]	+ [477]

Priority targets that were not only relevant for genomic instability, but also relevant for other aspects of cancer's biology (i.e., anticarcinogenic) were noted as having complementary effects (+). Those targets that were found to have procarcinogenic actions were noted as having contrary effects (−). In instances where reports on relevant actions in other aspects of cancer biology were mixed (i.e., reports showing both procarcinogenic potential and anticarcinogenic potential), the designation (±) was used. Finally, we indicate (0) in instances where no literature support was found to document the relevance of a target in a particular aspect of cancer's biology.

**Table 2:** Cross-validation for priority approaches against genomic instability.

Other cancer hallmarks	Priority approaches for genomic instability						
	Vitamin D	Vitamin B	Selenium	Carotenoids	PARP inhibitor	Resveratrol	Isothiocyanates
Sustained proliferative signaling	+ [478]	± [259], [426]	± [357], [479]	+ [480], [481], [482]	+ [358], [483], [484]	+ [354], [355]	+ [485], [486]
Tumor-promoting inflammation	+ [487], [488]	+ [393], [394]	− [395], [396], [397]	+ [398], [399]	+ [400], [401]	+ [402], [489]	+ [403], [404]
Evasion of anti-growth signaling	+ [490], [491], [492]	0	+ [493], [494]	± [366], [495]	+ [358]	+ [496], [497]	+ [360], [361], [364]
Resistance to apoptosis	+ [380]	0	+ [378]	+ [377]	+ [498]	+ [382]	+ [379]
Replicative immortality	+ [369], [370], [374]	0	+ [182]	+ [369], [372], [373]	+ [368], [499]	+ [375], [376]	+ [364], [371]
Dysregulated metabolism	0	0	0	+ [500]	0	+ [268], [501], [502], [503], [504], [505], [506], [507], [508]	+ [364], [383], [509]
Immune system invasion	+ [406]	0	0	+ [405]	0	+ [407], [510], [511], [512], [513]	0
Angiogenesis	+ [514]	± [408], [409], [410], [412], [414]	± [411], [413], [415], [417]	+ [416]	+ [418]	+ [515], [516]	+ [419]
Tissue invasion and metastasis	+ [517]	+ [425], [426]	+ [351], [427], [428]	+ [429], [431], [518]	+ [484]	+ [519]	+ [363], [420], [430]
Tumor microenvironment	+ [520]	0	+ [395]	+ [521]	+ [476]	+ [522]	+ [523]

Approaches that are not only relevant for genomic instability, but also relevant for other aspects of cancer's biology were noted as having complementary effects (+). Those approaches that were found to have procarcinogenic actions were noted as having contrary effects (−). In instances where reports on relevant actions in other aspects of cancer biology were mixed, the designation (±) was used. Finally, we indicate (0) in instances where no literature support was found to document the relevance of an approach in a particular aspect of cancer's biology.
